# Investigation of the Interactions between the Hydrophobic Cavities of Cyclodextrins and Pullulanase

**DOI:** 10.3390/molecules16043010

**Published:** 2011-04-07

**Authors:** Bo Yu, Jinpeng Wang, Huanxin Zhang, Zhengyu Jin

**Affiliations:** 1 State Key Laboratory of Food Science and Technology, School of Food Science and Technology, Jiangnan University, 1800 Lihu Road, Wuxi 214122, China; 2 Food Science Department, Jiangsu Animal Husbandry and Veterinary College, Taizhou 225300, China

**Keywords:** hydrophobic cavity, cyclodextrin, pullulanase, complex, fluorescence spectra

## Abstract

The effects of cyclodextrins and derivatives on the activity and structure of pullulanase were investigated in this study. Our results showed that cyclodextrins and derivatives decreased the activity of pullulanase. This decrease was attributed to the interaction between the hydrophobic cavities of cyclodextrins and pullulanase. The hydrophobic cavity was confirmed to encapsulate the groups of pullulanase molecules by the addition of competitive guests. The results obtained from fluorescence spectroscopy analysis showed that β-CD showed more efficient interactions with pullulanase molecules and the side chain groups of cyclodextrin significantly prevented the interaction between the hydrophobic cavities of β-CD and pullulanase molecules. These findings suggest that the geometric dimension of hydrophobic cavities was crucial for matching between cyclodextrins and pullulanase and steric hindrance caused by side chains led to the decrease of the interaction.

## 1. Introduction

Cyclodextrins (CDs) are a family of cyclic oligosaccharides composed of glucopyranose units linked by α-(1, 4)-glycosidic bonds to form a cylindric structure. On account of their hydrophobic cavities which could include or incorporate hydrophobic compounds, CDs have been used as host molecules, solubilizing agents, inhibitor blocking agents and molecular chelating agents [1,2].

Studies on CDs-enzyme supermolecular systems have given surprising results, for example, CDs were employed as additives to enhance reaction rates by increasing the solubility of hydrophobic substrates or encapsulating enzymes. On the contrary, they also serve as inhibitors, since CDs could form inclusion complexes with functional groups of enzymes such as aromatic amino acid residues [3,4,5,6]. Previous researches have described the inhibition of CDs on the α-amylase family. CDs were competitive inhibitors for both plant and microbial β-amylases [7,8] and inhibited cyclodextrin mixed type glycosyltransferases [9,10]. Hydrolysis of starch by the amyloglucosidases was also inhibited by β-cyclodextrin (β-CD) [11,12].

Pullulanase (EC 3.2.1.41) is a member of the α-amylase family that specifically cleaves α-(1,6)-glycosidic bonds in amylopectin, pullulan and glycogen. In the CDs-pullulanase system, CDs were verified to be competitive inhibitors [13,14]. Inhibition of β-CD on pullulanase depended on concentration and tended to be enhanced with the increase of β-CD [15]. However, there are not only hydrophobic cavities but also many hydroxyl groups in CDs. Both of them could interact with other molecules by hydrogen bonding, hydrophobic interactions and van der Waals forces. More detailed information about the activity and structural features of pullulanase in the presence of different CDs contributes to reflect the actual interaction between CDs and pullulanase. The aim of our study was to investigate the effects of hydrophobic cavities of CDs on pullulanase by exploring activity and microenvironment changes.

## 2. Results and Discussion

### 2.1. Inclusion Hypothesis between β-CD and Pullulanase Molecules

**Figure 1:**
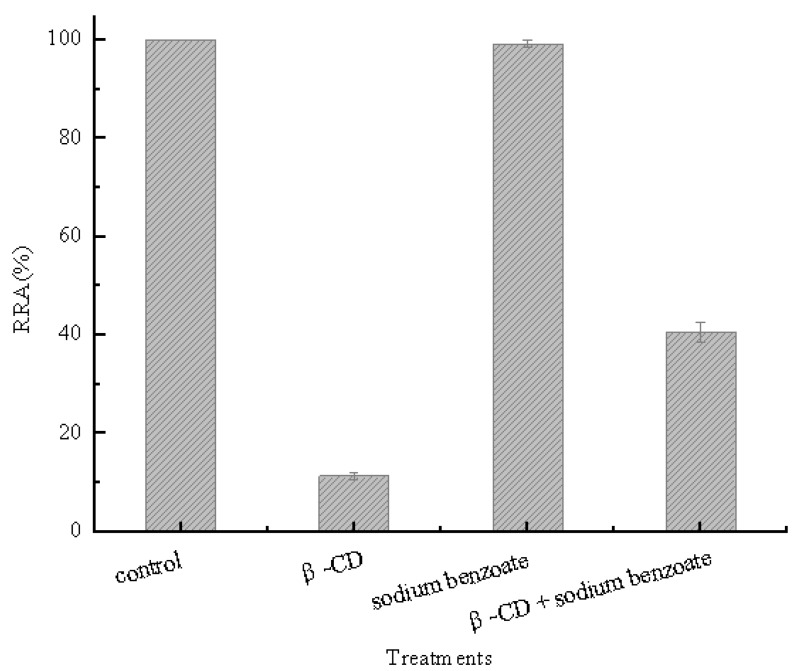
Effects of hydrophobic cavities on pullulanase activity.

β-CD could easily form inclusion complexes with sodium benzoate [16]. Thus, this substance was employed as indicator for our inclusion hypothesis. Sodium benzoate and β-CD (10 mM) were incubated overnight and then added into a pullulanase reaction system. Figure 1 shows that the activity of pullulanase decreased by approximately 90% when β-CD was added into the solution, while the activity of pullulanase remained at more than 40% when mixtures containing β-CD and sodium benzoate were added into the reaction system.

This indicated that sodium benzoate prevented the inactivation of pullulanase in the presence of β-CD. The prevention was attributed to competitive inclusion among hydrophobic cavities of β-CD, the benzyl structure of sodium benzoate and groups of pullulanase. These results suggest that hydrophobic cavities of β-CD formed inclusion complexes with some groups of pullulanase molecules.

### 2.2. Effect of Sizes of Hydrophobic Cavities on Pullulanase Activity

**Figure 2:**
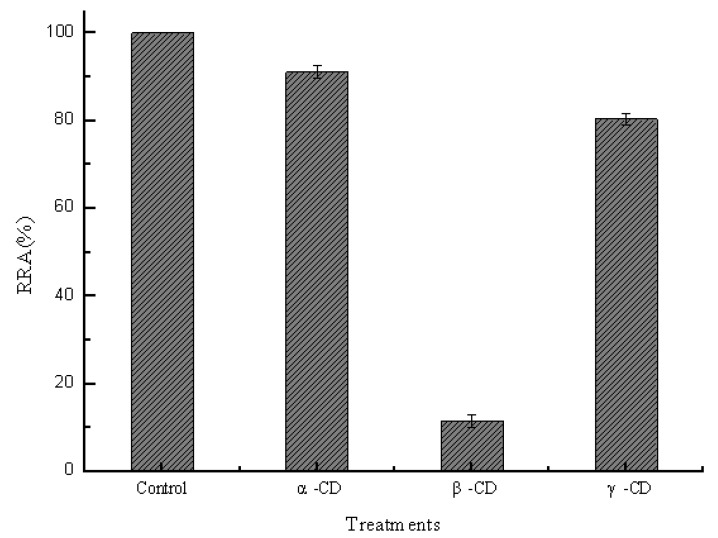
Effects of geometric dimension of hydrophobic cavities on the pullulanase activity.

In order to study the effects of hydrophobic cavities on pullulanase, the same concentrations of different CDs (10 mM) were added into a reaction system containing 0.2 μM pullulanase. Figure 2 shows that the activity of pullulanase was decreased by approximately 10%, 90% and 20% in the presence of α-CD, β-CD and γ-CD, respectively. The cavity diameters are 0.47–0.53, 0.6–0.65 and 0.75–0.83 nm for α-CD, β-CD and γ-CD, respectively, while the corresponding exterior diameters are 1.46 ± 0.04, 1.54 ± 0.04, 1.75 ± 0.04 nm for three common cyclodextrins. They possess truncated cone-shaped cavities which volumes in 1 mol are approx 104 mL, 157 mL and 256 mL, respectively [1]. Generally, complex formation between host (CDs) and a guest depend on size or shape of hydrophobic cavities. Thus, it was established that β-cyclodextrins molecules were of sufficient size and appropriate hydrophobic cavities for the formation of inclusion complexes with pullulanase molecules. 

**Figure 3:**
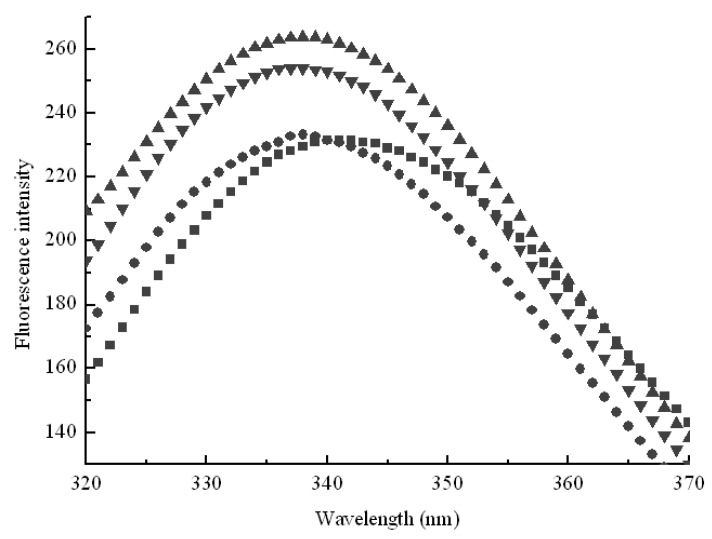
Effects of geometric dimension of hydrophobic cavities on intrinsic fluorescence of pullulanase in the absence of CDs (■) and in the presence of α-CD (●), β-CD (▲), γ-CD (▼). Each data point was the mean of three replicates.

In order to further explore effect of hydrophobic cavities size on pullulanase, variations of pullulanase structure were detected by fluorescence spectra, which was an effective technique to follow tertiary structure transitions in enzymes. As shown in Figure 3, the fluorescence intensity increased with the addition of CDs. Generally, the intrinsic fluorescence of protein derives from aromatic amino acids such as tryptophan, phenylalanine and tyrosine. Tryptophan residues are usually used as endogenous probes to reveal the change of tertiary structure and microenvironment of enzymes [17]. The results obtained from fluorescence spectra indicated that CDs formed inclusion complexes with tryptophan residues to protect the fluorescing singlet state. Moreover, hydrophobic cavities of CDs afford an apolar surrounding for tryptophan to enhance fluorescence quantum efficiencies. Figure 3 shows that the fluorescence intensity increased in sequence β-CD > γ-CD > α-CD, which corresponded with the decrease of pullulanase activity as shown in Figure 2. The results provided the evidence that β-CD showed more sufficient to interact with pullulanase molecules due to its geometric dimension. The maximum fluorescence intensity also exhibited blue shift (from 340 nm to 338 nm) with the addition of β-CD, as shown in Figure 3.

This indicated that β-CD changed the microenviroment around pullulanase and the native conformation gradually became loose. Some tryptophans buried inside pullulanase were exposed to a more apolar environment and the hydrophobicity around tryptophan residues increased. The native structure of pullulanase gradually unfolded and β-CD intensified the dispersion of pullulanase aggregation and furthermore changed the microenvironment around pullulanase, which gave rise to the loss of biological activity. These results suggest that the interaction between CDs and pullulanase was closely related to the sizes of hydrophobic cavities.

### 2.3. Effect of Side Chain Groups of β-CD on the Interaction between Cavities and Pullulanase Molecules

**Figure 4:**
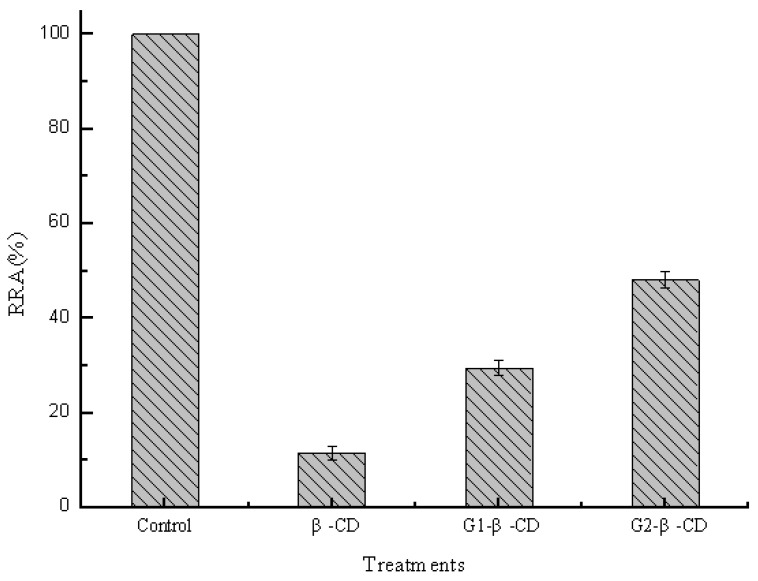
Effects of side chain groups of β-CD on pullulanase activity.

**Figure 5:**
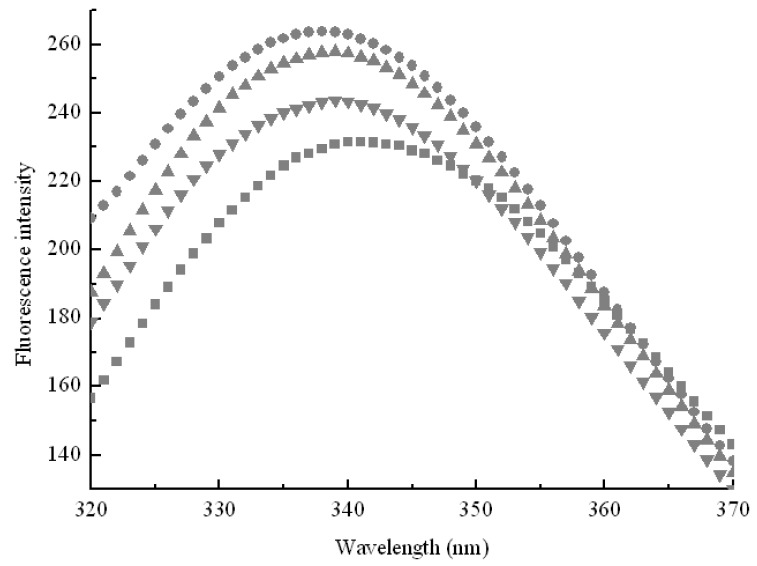
Effects of side chain groups of β-CD on intrinsic fluorescence of pullulanase in the absence of CDs (■) and in the presence of β-CD (●), G_1_-β-CD (▲), G_2_-β-CD (▼). Each data point was the mean of three replicates.

The interaction between branched cyclodextrins and pullulanase molecules was investigated by detecting the change of activities in pH 5.0 buffer. The same concentrations of different branched cyclodextrins (10 mM) were added into the reaction system containing 0.2 μM pullulanase. Figure 4 shows that the activities of pullulanase decreased by approximately 90%, 70% and 50% in the presence of β-CD, glucosyl-β-CD (G_1_-β-CD) and maltosyl-β-CD (G_2_-β-CD), respectively. It was apparent that side chain groups led to the decrease of the interaction between hydrophobic cavities of β-CD and pullulanase molecules. The weaker interaction between branched β-CDs and pullulanase may be due to a steric hindrance of side chain groups (maltose and glucuronylglucose), which grafted to parent rim by α-(1, 6)-glucosidic linkages. Moreover, the longer branch not only intensified the steric hindrance to prevent hydrophobic cavities of β-CD from interacting with pullulanase, but also gave rise to distortion of native hydrophobic cavities. It was further verified by the change of intrinsic fluorescence of pullulanase induced by branched CDs as shown in Figure 5, the fluorescence intensity increased in sequence β-CD > G_1_-β-CD > G_2_-β-CD and kept in accordance with the decrease of pullulanase activity. The fluorescence spectrum also exhibited blue shift of the maximum but slightly changed in the presence of side chain groups compared to native rim. This spectrum shift indicated that the hydrophobicity around tryptophan residues changed, since polarity for native β-CD induced by side chain groups. Thus, it was apparent that side chain groups led to decrease the interaction between hydrophobic cavities of β-CD and pullulanase molecules.

## 3. Experimental

### 3.1. Materials and Chemicals

Pullulanase from *Bacillus acidopullulyticus* was purchased from Genencor Inc. (Wuxi, China). CDs were purchased from Sigma-Aldrich Trading Co., Ltd (Shanghai, China). All other chemicals and reagents were of reagent grade.

### 3.2. Purification of Pullulanase from Bacillus Acidopullulyticus

Pullulanase was dialyzed overnight against acetate buffer (20 mM, pH 5.0) to remove a great many saccharides and then concentrated against polyethylene glycol 20,000 at 4 °C. The concentrated enzyme was further purified through HiPrep16/10DEAEFF ion exchange chromatography and HiPrep16/60Sephacryl S-200HR chromatography. All subsequent chromatographic steps were performed using an Akta Purifier 10 system (Pharmacia Amersham Biotech, Sweden). The purified pullulanase was analyzed by sodium dodecyl sulphate-polyacrylamide gel electrophoresis and was certified a single band.

### 3.3. Pullulanase Activity Assays

Pullulanase activity was determined by measuring the amount of enzyme to release reducing sugars during incubation with pullulan. In this assay, the reaction mixture containing 0.5 mL of 1% (w/v) pullulan in 20 mM acetate buffer (pH 5.0) and 0.5 mL of enzyme sample was incubated at 50 °C for 30 min. The reducing sugar released was determined by Dinitrosalisyilic Acid method (DNS) [18]. One unit of pullulan activity was defined as the amount of enzyme that catalyzed the formation of 1 μmol of reducing sugars per minute. A V-1800 spectrophotometer (Mapada，Shanghai, China) was used for all assays. Relative residual activity (RRA) of pullulanase was defined as a percentage of activity of the pullulanase induced by different concentrations of β-CD relative to that of the enzyme sample without β-CD.

### 3.4. Fluorescence Spectroscopy

Fluorescence spectra were recorded using an F-7000 fluorescence spectrometer (Hitachi, Japan) at 295 nm (excitation wavelength, slit = 5.0 nm), 200–400 nm (emission wavelength, slit = 5.0 nm) and 12,000 nm/min of scanning speed.

## 4. Conclusions

This work successfully investigated effects of hydrophobic cavities of CDs on pullulanase by exploring activity and microenvironment changes. Inclusion hypothesis between β-CD and pullulanase molecules was confirmed by the addition of competitive guests. The change of intrinsic fluorescence spectroscopy provided the evidence for matching effect between geometric dimension of CDs and pullulanase molecules. Compared with other CDs, β-CD showed more capacity to interact with pullulanase molecules due to its appropriate geometric dimensions. Experimental data confirmed that side chain groups of branched CDs led to a decrease in the interaction between hydrophobic cavities and pullulanase molecules and longer side chain further intensified the hindrance effects. The present results are important supplements to our understanding of the interactions between CDs and pullulanase. These findings suggest that structure of CDs should be taken into consideration in the further research of cyclodextrin-protein interactions.
